# Relationships between biomarkers of cartilage, bone, synovial metabolism and knee pain provide insights into the origins of pain in early knee osteoarthritis

**DOI:** 10.1186/ar3246

**Published:** 2011-02-14

**Authors:** Muneaki Ishijima, Taiji Watari, Kiyohito Naito, Haruka Kaneko, Ippei Futami, Kaori Yoshimura-Ishida, Akihito Tomonaga, Hideyo Yamaguchi, Tetsuro Yamamoto, Isao Nagaoka, Hisashi Kurosawa, Robin A Poole, Kazuo Kaneko

**Affiliations:** 1 Department of Medicine for Motor Organ, Juntendo University Graduate School of Medicine, 2-1-1, Hongo, Bunkyo-ku, Tokyo 113-8421, Japan; 2 Department of Orthopaedics, Juntendo University School of Medicine, 2-1-1, Hongo, Bunkyo-ku, Tokyo 113-8421, Japan; 3 Sportology Center, Juntendo University Graduate School of Medicine, 2-1-1, Hongo, Bunkyo-ku, Tokyo 113-8421, Japan; 4 Department of Host Defense and Biochemical Research, Juntendo University Graduate School of Medicine, 2-1-1, Hongo, Bunkyo-ku, Tokyo 113-8421, Japan; 5 Department of Orthopaedic Surgery, Juntendo University Shizuoka Hospital, 1129 Nagaoka, Izunokuni-shi, Shizuoka 410-2295, Japan; 6 Total Technological Consultant Co., Ltd., 1-20-2, Ebisunishi, Shibuya-ku, Tokyo 150-0021, Japan; 7 Tana Orthopaedic Surgery, 15-7, Tanacho, Aoba-ku Yokohama, Kanagawa 227-0064, Japan; 8 Professor Emeritus, Department of Surgery, McGill University, 1650 Cedar Ave., Montreal, Quebec, H3G 1A4, Canada

## Abstract

**Introduction:**

We tested the hypothesis that there exist relationships between the onset of early stage radiographically defined knee osteoarthritis (OA), pain and changes in biomarkers of joint metabolism.

**Methods:**

Using Kellgren-Lawrence (K/L) grading early radiographic knee OA (K/L 2) was detected in 16 of 46 patients. These grades (K/L 1 is no OA and K/L 2 is early OA) were divided into two groups according to the presence or absence of persistent knee pain. Sera (s) and urines (u) were analysed with biomarkers for cartilage collagen cleavage (sC2C and uCTX-II) and synthesis (sCPII), bone resorption (uNTx) and synovitis (hyaluronic acid: sHA).

**Results:**

sCPII decreased and sC2C/sCPII, uCTX-II/sCPII and sHA increased with onset of OA (K/L 2 versus K/L 1) irrespective of joint pain. In contrast, sC2C and uCTX-II remained unchanged in early OA patients. Of the patients with K/L grades 1 and 2 sC2C, sCPII, sHA, uNTX and uCTX-II were all significantly increased in patients with knee pain independent of grade. Among the K/L grade 2 subjects, only uCTX-II and uCTX-II/sCPII were increased in those with knee pain. In grade 1 patients both sC2C and sCPII were increased in those with knee pain. No such grade specific changes were seen for the other biomarkers including sHA.

**Conclusions:**

These results suggest that changes in cartilage matrix turnover detected by molecular biomarkers may reflect early changes in cartilage structure that account directly or indirectly for knee pain. Also K/L grade 1 patients with knee pain exhibit biomarker features of early OA.

## Introduction

Pain is the most prominent and disabling symptom of knee osteoarthritis (OA) and is an increasingly important public health problem [1-4]. Pain is the major reason why individuals seek medical attention from early-through end-stage knee OA, the treatment of which in advanced disease commonly includes joint replacement. Pain is also a major determinant for the loss of joint function. Given the lack of any disease-modifying drugs (DMORDs) for the treatment for knee OA, present treatments are essentially for knee pain [5]. Despite its importance, much remains unknown about the nature, causes, and natural history of OA joint pain.

The gold standard for assessing joint damage is still the plain radiograph. However, this method only provides a historical view of the skeletal damage that has already occurred. Radiography is relatively insensitive, and does not allow for the early detection of pathological changes in joint tissues and early joint damage. In addition, often weak associations have been reported between pain and radiographic change [6].

There is an urgent need for an improved understanding of the origins of joint pain and tests that allow for the evaluation of treatment responses designed to arrest joint destruction and control pain [7]. In joint diseases, there is a loss of the normal balance between the synthesis and degradation of the molecules that provide the articular cartilage with its biochemical and functional properties [8,9]. Concomitantly, changes occur in the metabolism of the synovium [10] and in the turnover of the subchondral bone [11]. Biomarkers, in addition to other imaging technologies such as magnetic resonance imaging (MRI), are candidates that are now being used to detect and monitor cartilage, bone turnover and synovial metabolism for critical assessment of the pathophysiological processes that lead to joint failure and pain in OA patients [8,12-15].

This study of early knee OA and joint pain was designed to test the hypothesis that there exist interrelationships between the onset of early stage radiographically defined knee OA, the presence and absence of knee pain and changes in skeletal and synovitis biomarkers of tissue turnover and joint inflammation, respectively, that can be measured in body fluids. Based on earlier work with tissue specific biomarkers we believe that their use may aid in the understanding of the source(s) of knee pain.

Prior studies of OA knee pain involving biomarkers are limited but results have been encouraging. Briefly, elevations of serum cartilage oligomeric matrix protein (COMP) have shown an association with knee pain but not for the cartilage collagen biomarker CTX-II [16], the latter being derived mainly from calcified cartilage such as is found at the osteo-chondral junction [17] and in osteophytes.

Since it is known that structural changes in OA joints involving articular cartilage, as revealed by Kellgren and Lawrence grading [18] and MRI [19], are closely associated with knee pain we decided to examine other skeletal biomarkers of cartilage and bone turnover in addition to CTX-II and hyaluronan, a marker of synovitis [20,21].

Five commercial biomarker assays were used in these analyses: serum cartilage type II collagen cleavage by collagenase (sC2C) [8,15]; urinary cartilage type II collagen C-telopeptide (uCTX-II) [22]; serum cartilage type II procollagen carboxy propeptide (sCP II), which is cleaved from cartilage type II procollagen following the release of newly synthesized procollagen into the matrix [8,15,23]; urinary bone N-terminal crosslinking telopeptide of type I collagen (uNTx), a biomarker of bone resorption [24]; and serum hyaluronic acid (sHA) for synovitis [25,26], all of which have been used to study OA pathology [25-31].

## Materials and methods

### Ethics approval

The study protocol was approved by the institutional review board of Juntendo University. We received a written consent from each of the patients enrolled in this study.

### Patients, radiography and pain

A total of 46 patients with a Kellgren-Lawrence (K/L) grade of 1 or 2 were enrolled in this study among the patients who visited the hospitals from September 2007 to March 2008. Patients with knee pain had complained of pain in the medial femorotibial compartment of the studied knee on most days of the month prior to examination and removal of body fluids. Patients had not experienced any traumatic episodes during this period. Knee pain was assessed using a visual analogue scale (VAS, 0 to 100). The subjects were defined as those without pain if they indicated a VAS score of zero for this same period. Conversely, subjects were defined as having pain if they indicated a score of more than zero, and they fulfilled the criteria of knee OA of the medial femorotibial joint as defined by the American College of Rheumatology (ACR) criteria [32]. Standing, extended and antero-posterior view, and lateral and skyline view radiographs were taken at the first visit. An antero-posterior view radiograph was performed according to the method reported previously [33]. The staging of knee OA based on radiographic examination was assessed using K/L grading [34]. As with the traditional radiographic definition for OA, the subjects with a K/L grade of 1 were diagnosed as having no detectable knee OA. Those with a K/L grade of 2 were diagnosed as having early knee OA. All radiographs were taken by experienced technicians and were scored independently by two readers who were blinded to the clinical information. Both intra- and inter-observer reproducibility rates were good (interclass correlation coefficient (ICC): 0.97 (95% confidence interval (CI): 0.70 to 0.97) and 0.95 (95% CI: 0.93 to 0.98), respectively).

### Biomarker analyses

Both serum and urine samples were obtained from all patients on the day that pain and function were assessed, and radiographs taken. The non-fasted second void urine samples and non-fasted morning blood samples were collected for serum analyses. The urine and serum samples were stored at -80°C until analysed.

The biomarkers used were sC2C, sCPII, sHA, uCTX-II, and uNTx. uCTX-II and uNTx values were corrected for urine creatinine concentration.

All assays, including measurement of urine creatinine levels, involved an ELISA format and were supplied with the following specifiations: uCTX-II (CartiLaps; Nordic Bioscience, Herlev, Denmark: intra-assay and interassay variation each less than 7%); uNTx (Osteomark; OrthoClinical Diagnostics, Rochester, NY, USA: intra-assay and interassay variations less than 5% and 7%, respectively); sCPII and sC2C (IBEX Pharmaceuticals Inc., Montreal, QC, Canada: intra-assay and interassay variation for sCP II and sC2C were less than 10% and 11%, respectively); sHA (Chugai Diagnostics, Tokyo, Japan: intra-assay and interassay variation were each less than 5%). The measurement of urinary creatinine concentration was performed by a peroxidase-coupled kinetic enzymatic procedure (Kainos, Tokyo, Japan) [35].

### Statistics

The statistical analyses were conducted using the SPSS version 17.0 for Windows software program (SPSS, Chicago, IL, USA). As the distribution of these biomarkers was found to be positively skewed, a logarithmic transformation (natural log (Ln)) was therefore applied to these biomarkers to obtain an approximately normal distribution. This was evaluated by the Kolmogorov-Smirnoc test before performing the statistical analyses. The serum and urinary levels of these biomarkers were adjusted for age, gender and body mass index (BMI) for comparison using parametric comparisons analysis of variance (ANOVA). The Bonferroni correction for multiple comparisons was applied. Significant differences were evaluated if ANOVA was significant. A *P*-value of less than or equal to 0.05 was considered to be statistically significant.

## Results

### Patients

Of the 46 patients, 30 had a K/L grade of 1 (female/male ratio: 19/11) and 16 (female/male: 13/3) had a K/L grade of 2 (Tables 1 and 2). Sixteen patients had knee pain (female/male ratio: 12/4), and the remaining 30 patients (female/male ratio: 20/10) had no knee pain (Tables 1 and 3). The 30 patients without knee pain consisted of 22 (female/male ratio: 13/9) with a K/L grade of 1 and 8 (female/male ratio: 7/1) with a K/L grade of 2 (Table 1 and 4). Eight (female/male ratio: 6/2) of the 16 patients with knee pain had a radiographic grade of 1, and the remaining half (female/male ratio: 6/2) had a K/L grade of 2 (Tables 1 and 4).

**Table 1:** Basal characteristics of the subjects in the study

		Total	K/L 1	K/L 2	*P-*value
n		46	30	16	
Pain (-/+)		30/16	22/8	8/8	
Gender (F/M)		32/14	19/11	13/3	
Age		67.5	67.4	68.0	0.31
(y)		7.1 (65.3 to 69.8)	6.8 (64.8 to 69.9)	8.3 (62.4 to 73.6)	(-7.01 to 2.24)
BMI		24.9	24.5	26.1	0.37
(kg/m^2^)		4.4 (23.6 to 26.3)	4.3 (22.9 to 26.1)	4.6 (23.0 to 29.2)	(-4.74 to 1.49)
Pain VAS score (0 to 100)	Pain (-)	0	0	0	
		0 (0 to 0)	0 (0 to 0)	0 (0 to 0)	
	Pain (+)	35.1	37.5	25.3	0.14
		12.9 (25.9 to 44.2)	12.2 (27.2 to 47.7)	14.0 (14.2 to 38.6)	(-0.21 to 2.66)

F, female; K/L, Kellgren-Lawrence grade; M, male. Data are presented as the mean and SD (95% confidence interval (95% CI) of the mean).

**Table 2:** Biomarker levels in each subgroup of the subjects according to the radiographic classification

	Radiographic OA	Mean	SE	95% CI of the mean		*P-*value	95% CI for difference
				Lower	Upper		
sC2C	K/L1	5.56	0.04	5.49	5.64	0.06	-0.09 to 0.30
Ln (pmol/ml)	K/L2	5.42	0.07	5.29	5.55		
sCPII	K/L1	7.53	0.06	7.38	7.64	<0.01*	0.26 to 0.78
Ln (ng/ml)	K/L2	6.99	0.11	6.77	7.21		
uCTX-II/Cr	K/L1	5.25	0.13	4.99	5.52	0.15	-0.94 to 0.15
Ln (ng/μmol creatinine)	K/L2	5.65	0.23	5.19	6.11		
sC2C/sCPII	K/L1	0.14	0.02	0.10	0.18	<0.01*	-0.21 to -0.04
	K/L2	0.26	0.04	0.19	0.34		
uCTX-II/sCPII	K/L1	0.11	0.06	-0.01	0.22	<0.01*	-0.68 to -0.22
	K/L2	0.56	0.10	0.37	0.75		
uNTx/Cr	K/L1	3.79	0.10	3.60	3.98	0.10	-1.47 to -0.59
Ln (ng/ml creatinine)	K/L2	3.46	0.16	3.13	3.79		
sHA	K/L1	2.97	0.12	2.74	3.21	<0.01*	-1.21 to -0.27
Ln (pmol/ml)	K/L2	3.71	0.20	3.31	4.11		

All analyses were adjusted for age, gender, and body mass index. The urinary biomarkers were corrected for creatinine. The number of subjects with K/L grade 1 was 30, while that with K/L grade 2 was 16. *P-values less than or equal to 0.05 are statistically significant. 95% CI, 95% confidence interval; C2C, cartilage collagen type II cleavage; CPII, cartilage type II collagen carboxy propeptide; CTX-II, type II collagen C-telopeptide; HA, hyaluronic acid; K/L, Kellgren-Lawrence grade; Ln, natural log; NTx, N-terminal crosslinking telopeptide of type I collagen; SE, standard error of the mean; s, serum; u, urine.

**Table 3:** Biomarker levels in each subgroups according to the presence or absence of knee pain

	Pain	Mean	SE	95% CI of the mean		*P-v*alue	95% CI for difference
				Lower	Upper		
sC2C	-	5.47	0.04	5.40	5.55	0.01*	-0.35 to -0.05
Ln (pmol/ml)	+	5.67	0.06	5.55	5.79		
sCPII	-	7.28	0.07	7.14	7.43	0.03*	-0.62 to -0.04
Ln (ng/ml)	+	7.62	0.12	7.37	7.86		
uCTX-II/Cr	-	5.14	0.12	4.90	5.38	<0.01*	-1.30 to -0.34
Ln (ng/μmol creatinine)	+	5.96	0.20	5.56	6.37		
sC2C/sCPII	-	0.19	0.02	0.14	0.23	0.40	-0.05 to 0.13
	+	0.15	0.04	0.07	0.22		
uCTX-II/sCPII	-	0.19	0.07	0.06	0.32	0.25	-0.42 to 0.12
	+	0.34	0.11	0.12	0.57		
uNTx/Cr	-	3.60	0.09	3.41	6.79	0.05*	-0.76 to 0.03
Ln (ng/ml creatinine)	+	3.98	016	3.66	4.30		
sHA	-	3.03	0.12	2.78	3.28	0.04*	-1.03 to -0.03
Ln (pmol/ml)	+	3.55	0.21	3.13	3.98		

All analyses were adjusted for age, gender, and body mass index. The urinary biomarkers were corrected for creatinine. The number of subjects with knee pain was 30, while the number without knee pain was 16. *P-values less than or equal to 0.05 are statistically significant.

95% CI, 95% confidence interval; C2C, cartilage collagen type II cleavage; CPII, cartilage type II collagen carboxy propeptide; CTX-II, type II collagen C-telopeptide; HA, hyaluronic acid; K/L, Kellgren-Lawrence grade; Ln, natural log; NTx, N-terminal crosslinking telopeptide of type I collagen; s, serum; SE, standard error of the mean; u, urine.

**Table 4:** Biomarker levels in each subgroups according to both the radiographic classification and knee pain

	Radiographic OA	Pain	Mean	SE	95% CI of the mean		*P-*value	95% CI for difference
					Lower	Upper		
**sC2C**	K/L 1	-	5.49	0.04	5.40	5.57	0.02*	-0.19 to -0.04
Ln (pmol/ml)		+	5.75	0.07	5.61	5.91		
	K/L 2	-	5.43	0.07	5.29	5.57	1.00	-0.36 to 0.35
		+	5.44	0.11	5.21	5.66		
**sCPII**	K/L 1	-	7.38	0.07	7.24	7.52	0.01*	-0.83 to -0.10
Ln (ng/ml)		+	7.84	0.11	7.62	8.07		
	K/L 2	-	7.05	0.11	6.83	7.28	1.00	-0.44 to 0.71
		+	6.92	0.18	6.56	7.28		
**uCTX-II/Cr**	K/L 1	-	5.04	0.14	4.76	5.32	0.07	-1.48 to 0.03
Ln (ng/μmol creatinine)		+	5.77	0.22	5.32	6.22		
	K/L 2	-	5.38	0.23	4.92	5.83	<0.05*	-2.36 to -0.05
		+	6.56	0.36	5.82	7.29		
**sC2C**	K/L 1	-	0.15	0.03	0.10	0.20	1.00	-1.12 to 0.17
**/sCPII**		+	0.12	0.04	0.04	0.21		
	K/L 2	-	0.27	0.04	0.19	0.36	1.00	-0.17 to 0.26
		+	0.23	0.07	0.09	0.37		
**uCTX-II/sCPII**	K/L 1	-	0.09	0.06	-0.03	0.22	1.00	-0.39 to 0.29
		+	0.14	0.10	-0.06	0.35		
	K/L 2	-	0.42	0.10	0.21	0.63	<0.05*	-1.07 to 0
		+	0.95	0.17	0.62	1.29		
**uNTX/Cr**	K/L 1	-	3.71	0.11	3.48	3.93	1.00	-0.89 to 0.33
Ln (ng/ml creatinine)		+	3.99	0.18	3.62	4.35		
	K/L 2	-	3.33	0.18	2.96	3.70	0.73	-1.49 to 0.41
		+	3.87	0.29	3.27	4.47		
**sHA**	K/L 1	-	2.77	0.13	2.51	3.03	0.06	-1.40 to 0.01
Ln (pmol/ml)		+	3.46	0.21	3.04	3.89		
	K/L 2	-	3.66	0.21	3.24	4.09	1.00	-1.42 to 0.78
		+	3.98	0.34	3.29	4.67		

All analyses were adjusted for age, sex and body mass index. The urinary biomarkers were corrected for creatinine. *P-values less than or equal to 0.05 are statistically significant.

95% CI, 95% confidence interval; C2C, cartilage collagen type II cleavage; CPII, cartilage type II collagen carboxy propeptide; CTX-II, type II collagen C-telopeptide; HA, hyaluronic acid; K/L, Kellgren-Lawrence grade; Ln, natural log; NTx, N-terminal crosslinking telopeptide of type I collagen; s, serum; SE, standard error of the mean; u, urine.

### Biomarker analyses

#### According to K/L grade (Table 2)

The patients were divided into two groups according to the presence (K/L grade 2) or absence (K/L grade 1) of radiographic OA, and the characteristics of these two groups were examined by biomarker analyses. There were no significant differences for the cartilage collagen degradation markers sC2C and uCTX-II between K/L grade 1 and grade 2. sCPII, a cartilage collagen synthesis marker, was significantly reduced in K/L grade 2 compared to grade 1. No significant differences in uNTx, the bone resorption biomarker, were observed between K/L grade 1 and grade 2. But sHA was significantly increased in K/L grade 2 compared to grade 1. As ratios combining type II collagen degradation markers with type II collagen synthesis markers have previously also provided important insights into the balance between type II collagen degradation and synthesis, which is changed in OA [8,36], these ratios were also calculated. Both sC2C/sCPII and uCTX-II/sCPII were significantly increased in K/L grade 2 compared to grade 1 reflecting alterations in the balance between synthesis and degradation.

#### According to presence or absence of pain (Table 3)

Patients were divided into two groups according to the presence or absence of knee pain, and these two groups were evaluated by biomarker analyses. The levels of cartilage biomarkers sC2C, uCTX-II, sCPII and the synovitis biomarker sHA were all significantly increased in patients with knee pain compared to those without knee pain irrespective of K/L grade. In contrast, there was only a marginally (*P *= 0.05) significant difference for the bone biomarker uNTx in patients with or without knee pain irrespective of grade. The presence of pain did not influence the ratio of sC2C/sCPII nor that of uCTX-II/sCPII.

#### According to K/L grade with or without knee pain (Table 4)

The participants were further divided into four groups according to the presence (K/L 2) or absence (K/L 1) of both radiographic knee OA and knee pain. sC2C in K/L grade 1 patients with knee pain was significantly increased in comparison to the same grade group without knee pain. No such differences were seen in K/L grade 2 patients. sCPII in K/L grade 1 patients with knee pain was significantly increased in comparison to those with no pain in this grade. Again such differences were not seen in K/L grade 2. In contrast, uCTX-II in K/L grade 2 patients with knee pain was significantly increased in comparison to those in K/L grade 2 without knee pain. Such pain-related differences were not seen in K/L grade 1 patients. No significant differences in sC2C/sCPII were observed between sub-groups with and without pain for either grade. uCTX-II/CPII in K/L 2 patients with knee pain was significantly increased in comparison to those in K/L grade 2 without knee pain. No such pain-related differences were seen for uCTX-II/CPII in grade 1 patients. In contrast to the cartilage biomarkers, there were no significant differences in uNTx or sHA between sub-groups with and without pain for either grade.

## Discussion

We set out to determine with skeletal biomarkers and a synovitis biomarker whether we could identify early systemic differences in cartilage, bone and synovial metabolism that may be associated with joint pain in K/L grade 1 (supposedly normal) patients and in early (K/L grade 2) knee OA. Previously reported changes in a reduction of sCPII [23] and an increase in sHA [21], as well as changes in the ratios of cartilage collagen degradation (C2C and CTX-II) and synthesis (CPII) markers [8,36] were again observed with the onset of OA (K/L grade 2 versus K/L grade1). Moreover, the present study also revealed that some of these biomarkers can detect pain-associated differences in patients irrespective of grade. The differences in K/L grade 1 involving pain-related changes in the cartilage biomarkers C2C and CPII correspond to the fact that although these grade 1 patients with knee pain may appear normal radiographically they often exhibit early cartilage lesions revealed by MRI [15]. Others have found that chondral defects in articular cartilages seen on MRI are associated with OA knee pain [19]. Biomarker changes, involving the biomarkers C2C and CPII, similar to those seen in our study in K/L grade 2 versus grade 1 have previously been observed [8,15]. Together these and our results indicate that the cartilage biomarkers C2C, CTX-II and CPII can be used to help detect early chondral lesions in knee OA and that changes in these biomarkers associated with the pathology of knee OA are also associated with knee pain.

As cartilage is aneural, it is not a tissue that can directly generate pain [4]. But changes in articulation caused by structural and associated changes in extracellular matrix turnover in articular cartilages, reflected by cartilage biomarkers [8,15,36], may result in the manifestation of pain in other joint tissues. This may be a consequence of alterations in joint mechanics resulting in structural changes elsewhere and/or the generation of joint debris that may cause a synovitis.

Subchondral bone, periostium, synovium, ligaments, and joint capsule are all richly innervated and contain nerve endings that may be the source of pain in OA patients [2,4,5]. The severity of the synovitis as detected by MRI has been reported to be associated with joint pain in the knee [37]. In agreement with this we observed an increase sHA in patients experiencing joint pain when all patients were examined but this pain-related increase was not seen within grades, being only marginal (*P *= 0.06) in grade 1, probably due to the smaller study population.

Subchondral bone has been proposed as a source of joint pain in knee OA [1,4,5]. We noted an increase in uNTx, the bone resorption marker, in the joint pain sub-group when all patients were analysed although once again, as with sHA, this was not observed in analyses of each K/L grade, probably for the same aforementioned reasons.

The interface between subchondral bone and articular cartilage is a site of potential remodeling in OA, as elsewhere intraarticularly, although it has attracted limited attention. The recent discovery that the biomarker CTX-II originates primarily from calcified cartilage at this site [17] is of special interest since this biomarker was increased in patients with joint pain and is also increased in K/L grade 2 patients with knee pain over those without pain. Thus changes at this osteochondral junction may account, in part at least, for the relationship of this biomarker to joint pain. As it is likely that it is also generated in endochondral ossification, which involves calcified cartilage remodeling, and this is also a component of osteophyte formation, further work is required to determine whether the changes in CTX-II may also reflect osteophyte remodeling.

It has been generally accepted that a K/L grade of 2 is the cut-off for defining radiographic knee OA features. It has also been suggested that since no clear consensus exists as to whether K/L grade 1 subjects also represent early OA, they should be treated as patients of separate grades [38]. In addition, the subjects with a K/L grade of 1 have been shown to have an increased risk for progression to a K/L grade of 2 [38]. The results of the current study suggest that those patients with a K/L grade of 1 should, if knee pain is present, also be included as a separate group and one which may represent early OA in view of recent findings. Importantly, those with persistent knee pain but no prior diagnosis of OA have early OA in the majority of cases based on MRI and radiographic evidence [15]. This will be an important consideration for the future evaluation of biomarkers as a diagnostic tool [39,40].

The current study had some limitations. The interpretation of the results was limited by the small number of patients. However, the results usually showed convincing statistical significance. This investigation was a single-arm study and not a randomized trial. Therefore, the design may have introduced certain bias into the results. The serum and urine sample collections were not timed or fasted. Diurnal and activity related variation of some biomarkers have been reported [41,42]. But collection of second void urine samples was recommended in a previous study [42]. We included only Japanese samples in the analyses. As a result, our findings may not be applicable to other ethnic groups. Because we did not conduct detailed phenotyping of other joints, the contribution of other joints to the systemic levels of biomarkers cannot be addressed.

## Conclusions

In spite of these reservations, our results reveal that changes in cartilage matrix turnover detected by molecular biomarkers may reflect early changes in cartilage structure that account directly or indirectly for knee pain in both health and disease. The results also suggest that synovitis and bone remodeling may contribute to joint pain. The observation that K/L grade 1 patients with knee pain exhibit biomarker features of early knee OA is of special interest regarding the radiographic identification of early OA. This and previous but limited biomarker studies together suggest that biomarkers have value in helping identify the source of knee pain in patients with early OA and in the early detection of knee OA.

## Abbreviations

ACR: American College of Rheumatology; ANOVA: analysis of variance; C2C: cartilage type II collagen cleavage by collagenase; CI: confidence interval; COMP: cartilage oligomeric matrix protein; CP II: cartilage type II procollagen carboxy propeptide; CTX-II: cartilage type II collagen C-telopeptide; DMORDs: disease-modifying drugs; HA: hyaluronic acid; ICC: Interclass correlation coefficient; K/L grading: Kellgren-Lawrence grading; Ln: natural log; MRI: magnetic resonance imaging; NTx: N-terminal crosslinking telopeptide of type I collagen; OA: osteoarthritis; VAS: visual analogue scale.

## Competing interests

RP is a consultant to IBEX. The other authors declare that they have no competing interests.

## Authors' contributions

MI, KYI, TY, IN and HK conceived and designed the study. MI, TW, KN, HK, IF and AT undertook measurement of knee structures. MI, HK, IN, KK and RP had the major role in analysis and interpretation of the data, and contributed to drafting the report. HY supervised the statistical analysis. All authors have read and approved the final manuscript.
